# Dosimetry Prediction for Clinical Translation of ^64^Cu-Pembrolizumab ImmunoPET Targeting Human PD-1 Expression

**DOI:** 10.1038/s41598-017-19123-x

**Published:** 2018-01-12

**Authors:** Arutselvan Natarajan, Chirag B. Patel, Frezghi Habte, Sanjiv S. Gambhir

**Affiliations:** 1 Department of Radiology, School of Medicine, Stanford University, Stanford, CA United States; 2 Department of Bioengineering, Stanford University, Stanford, CA United States; 3 Materials Science & Engineering, Stanford University, Stanford, CA United States

## Abstract

The immune checkpoint programmed death 1 receptor (PD-1) expressed on some tumor-infiltrating lymphocytes, and its ligand (PD-L1) expressed on tumor cells, enable cancers to evade the immune system. Blocking PD-1 with the monoclonal antibody pembrolizumab is a promising immunotherapy strategy. Thus, noninvasively quantifying the presence of PD-1 expression in the tumor microenvironment prior to initiation of immune checkpoint blockade may identify the patients likely to respond to therapy. We have developed a ^64^Cu-pembrolizumab radiotracer and evaluated human dosimetry. The tracer was utilized to image hPD-1 levels in two subcutaneous mouse models: (a) 293 T/hPD-1 cells xenografted into NOD-scid IL-2Rγnull mice (NSG/293 T/hPD-1) and (b) human peripheral blood mononuclear cells engrafted into NSG bearing A375 human melanoma tumors (hNSG/A375). In each mouse model two cohorts were evaluated (hPD-1 blockade with pembrolizumab [blk] and non-blocked [nblk]), for a total of four groups (n = 3–5/group). The xenograft-to-muscle ratio in the NSG/293 T/hPD-1 model at 24 h was significantly increased in the nblk group (7.0 ± 0.5) compared to the blk group (3.4 ± 0.9), p = 0.01. The radiotracer dosimetry evaluation (PET/CT ROI-based and *ex vivo*) in the hNSG/A375 model revealed the highest radiation burden to the liver. In summary, we validated the ^64^Cu-pembrolizumab tracer’s specific hPD-1 receptor targeting and predicted human dosimetry.

## Introduction

The use of immune checkpoint (IC) blockade strategies, e.g. against programmed death-1 receptor (PD-1), PD-1’s ligand (PD-L1), and cytotoxic lymphocyte antigen-4 (CTLA4) is expanding in cancer immunotherapy^1^. Many preclinical studies have demonstrated that blockade of these ICs, particularly PD-1 and PD-L1, with monoclonal antibodies enhances tumor cell-specific T-cell activation, cytokine production, anti-tumor effector mechanisms, and clearance of tumor cells by the immune system^2–4^. These studies have led to accelerated FDA approval of several antibodies as a second-line therapy in advanced melanoma and non-small cell lung cancer (NSCLC).

**Figure 1:**
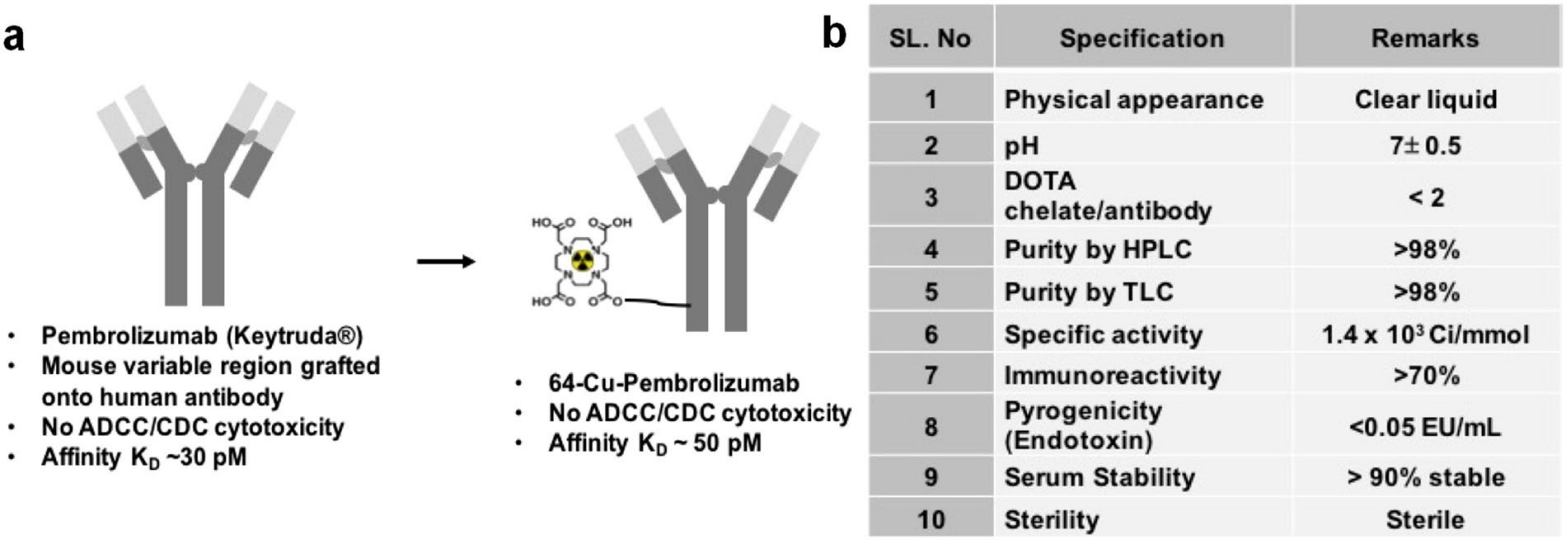
Radiotracer characterization. (**a**) Schematic diagram of pembrolizumab and ^64^Cu-pembrolizumab. (**b**) Summary of the quality assurance of ^64^Cu-pembrolizumab tracer used in the mouse studies.

Pre-clinical and clinical studies have shown that T-cell immune surveillance is controlled by the PD-1 pathway, which represents a major immune checkpoint that could be targeted for therapy^5^. PD-1 inhibition was tested in a clinical study of a variety of solid tumor types, and promising survival benefits were noted, including in melanoma and NSCLC^6^. Further, it has been suggested that PD-1 regulates tumor-specific T-cell expansion of tumor-infiltrating lymphocytes (TILs) in patients with melanoma^7^. Pembrolizumab is a high affinity (K_D_ = 29 pM) IgG4-κ humanized monoclonal antibody that is created by grafting the variable binding portion of a mouse anti-human PD-1 antibody onto a human isotype framework containing a stabilizing S228P mutation in the Fc region (see Fig. 1a).

Safety, tolerability, pharmacokinetics, and immunogenicity of pembrolizumab have been investigated in patients with advanced solid tumors including advanced melanoma and NSCLC^8,9^. In a cohort of 135 advanced melanoma patients undergoing anti-hPD-1 therapy, a durable response rate of greater than 50% was observed^8,10^. The ligands for PD-1 (PD-L1 and PD-L2) are constitutively expressed or can be induced in various tumors including melanoma^11–14^. The high expression of PD-L1 (and to a lesser extent, PD-L2) on tumor cells is correlated with poor prognosis in various cancer types, especially in NSCLC^15–17^.

hPD-1 receptor imaging could provide a non-invasive method to track and quantify the number of hPD-1-expressing TILs in the tumor microenvironment. In this regard, use of an immunoPET tracer may help to screen for cancer patients likely to respond to anti-PD-1 immunotherapy. In the present study, we have evaluated an anti-hPD-1 immunoPET tracer (^64^Cu-pembrolizumab) in two mouse models bearing human-derived cell lines, measured tracer residence time in clearance organs, and predicated human equivalent dosimetry.

## Results

### Synthesis and Quality Control of the immunoPET Tracer

HPLC-purified pembrolizumab was conjugated to DOTA-NHS (Do) with lysine groups of pembrolizumab (10:1 molar ratio) at pH 8.0, which yielded Do-pembrolizumab of 1–2 chelates per antibody (c/a), which was confirmed by MALDI-TOF mass spectrometry (Supplementary Fig. S1). This DOTA-conjugated antibody was further purified by SEC-2000-HPLC column and radiolabeling of ^64^Cu with Do-pembrolizumab yielded >70% ^64^Cu-pembrolizumab and provided >99% purity by SEC-2000-HPLC with 1.5 × 10^3^ Ci/mmol specific activity (Fig. 1b and Supplementary Fig. S2).

### ImmunoPET Images of NSG/293 T/hPD-1 Mouse Xenograft Model

**Figure 2:**
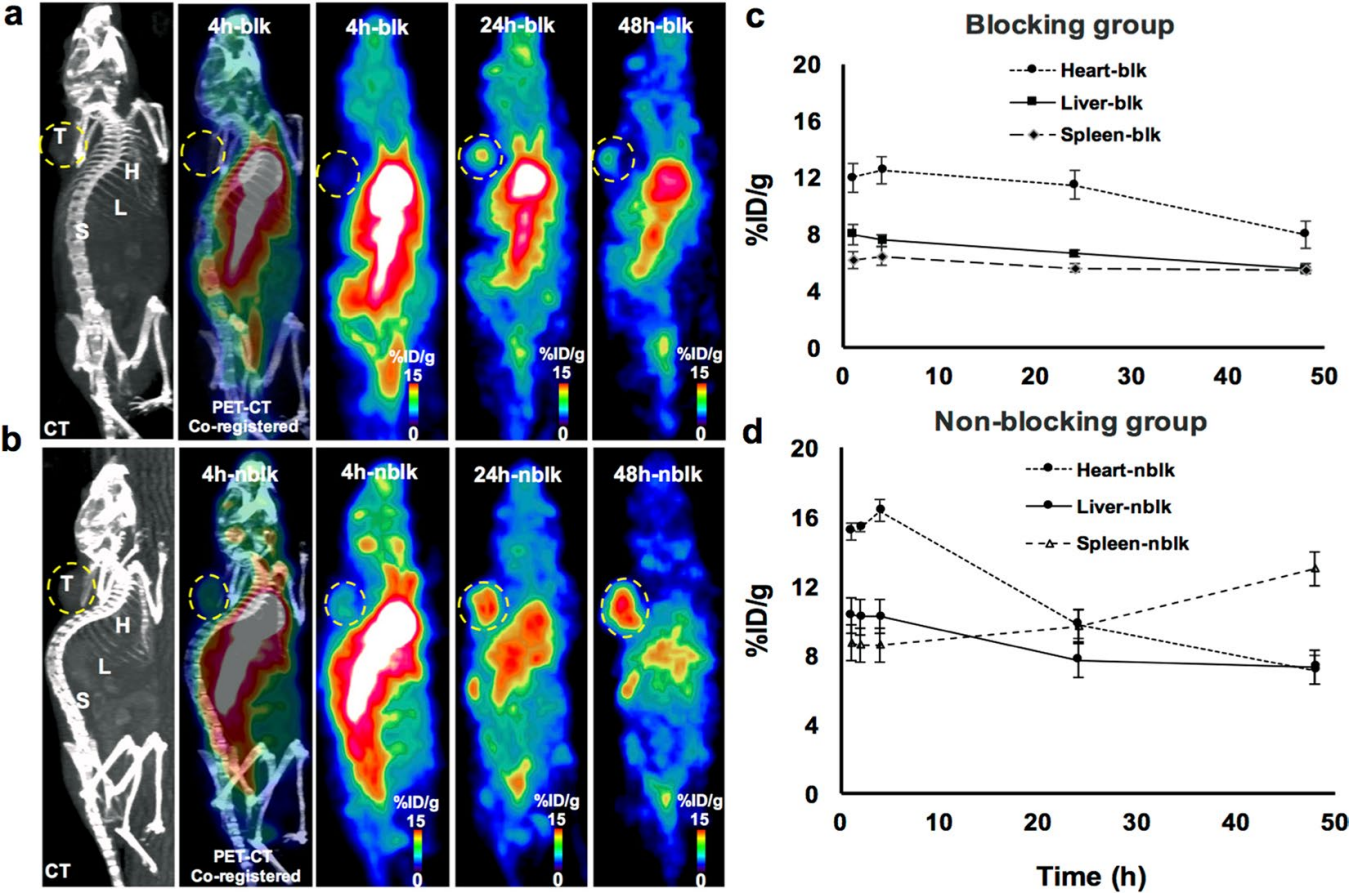
PET-CT image showing ^64^Cu-pembrolizumab immunoPET in NSG/293 T/hPD-1 mouse model. Representative PET images scanned at 4, 24, and 48 h post-injection of ^64^Cu-pembrolizumab tracer (7.4 MBq/200 μL) in **(a)** NSG/293 T/hPD-1-blk (hPD-1 pre-blocked with non-radioactive pembrolizumab and (**b**) NSG/293 T/hPD-1-nblk (non-blocking) mice. *L* liver, *H* heart, *X* xenograft, *S* spleen. (**c,d**) ImmunoPET signal was quantified in regions of interest in clearance organs (*H*, *L*, and *S*) from the images in (**a,b**). The PET signal was computed as mean ± standard deviation % ID/g at each time point over the 1–48 h post-injection window. Data are partial volume- and decay-corrected.

Because the athymic NSG/293 T/hPD-1 mice lacked intrinsic T cells and hPD-1, this model was chosen to avoid cross-reactivity with murine PD-1 during PET imaging. Figure 2 displays representative sagittal immunoPET images of NSG/293 T/hPD-1 mice (at 4, 24 and 48 h post-injection [p.i.]) bearing the hPD-1-expressing 293 T xenograft in the shoulder. These immunoPET images demonstrate that the ^64^Cu-pembrolizumab tracer specifically binds to hPD-1-expressing 293 T xenograft in the nblk group (Fig. 2b) compared to the blk group (Fig. 2a). The tracer clearance temporal profiles in the heart, liver, and spleen were quantified (ROI: mean ± SD % ID/g) and plotted against various time points (1–48 h) for each of the blk and nblk groups, and are presented in Fig. 2c and d, respectively. At 48 h, the heart/liver/spleen uptake in the blk and nblk groups was 7.9 ± 0.5/5.6 ± 0.4/5.5 ± 0.2 and 7.1 ± 0.8/7.3 ± 0.2/13.0 ± 0.8% ID/g, respectively.

### Tracer Uptake in Tumor and Clearance Organs of the NSG/293 T/hPD-1 Xenograft Mice and Biodistribution Study

**Figure 3:**
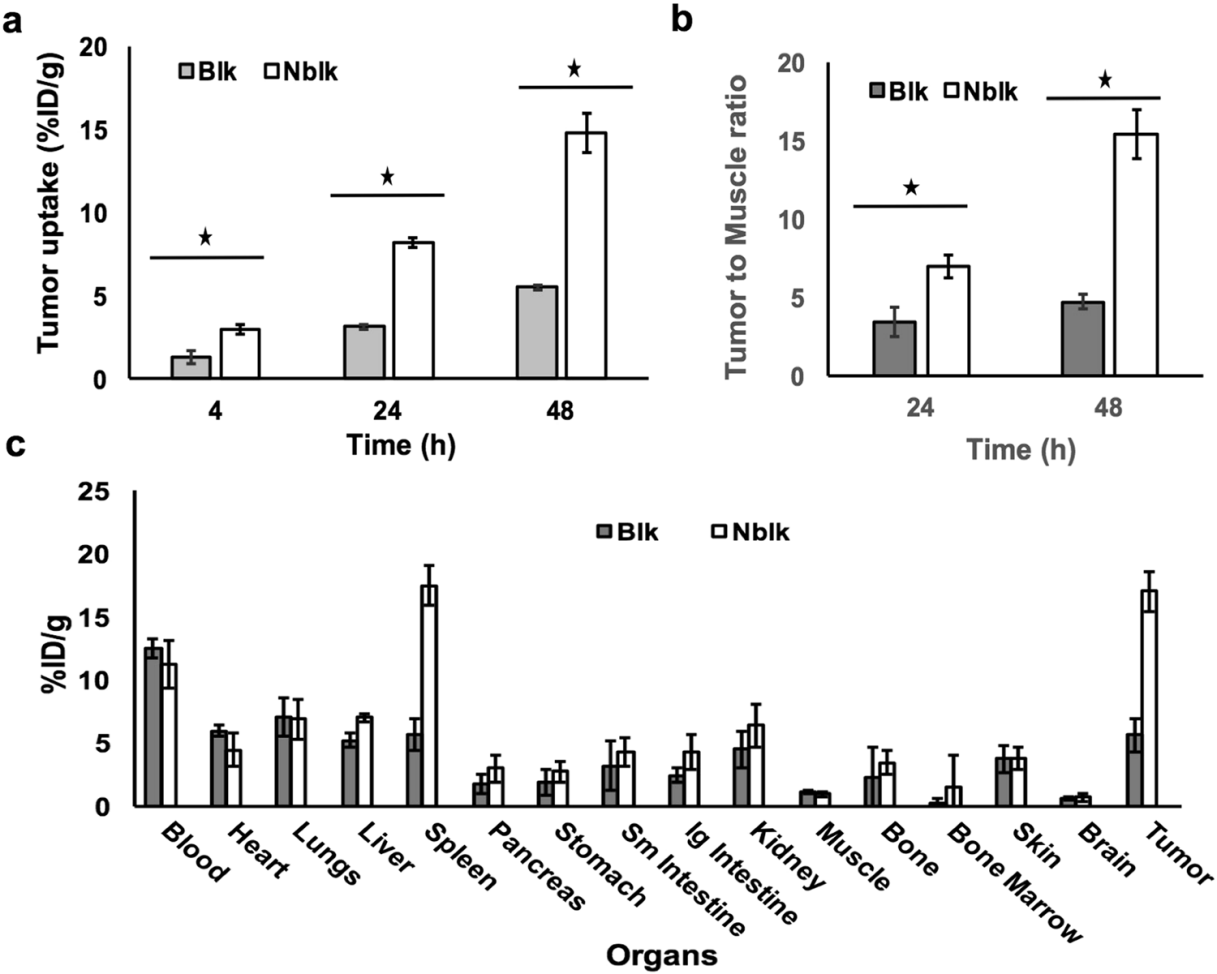
^64^Cu-pembrolizumab tracer uptake in NSG/293 T/hPD-1 from ROI and *ex vivo* biodistribution measurements. **(a)** Region of interest (ROI) quantification of immunoPET tracer signal (mean ± SD % ID/g) from the xenograft of NSG mice, NSG/293 T/hPD-1-blk (blocking: *n* = 4) and NSG/293 T/hPD-1-nblk (non-blocking: *n* = 4), after 4 24, and 48 h post-injection, * indicates p < 0.001. (**b**) Xenograft-to-muscle background ratios (XMR) were computed from ROIs at 24 and 48 h p.i. The XMR in NSG/293 T/hPD-1-nblk mice were significantly greater than in NSG/293 T/hPD-1-blk mice, p = 0.001. (**c**) Biodistribution profiles of immunoPET tracer in NSG/293 T/hPD-1-blk and NSG/293 T/hPD-1-nblk mice were compared after 48 h microPET-CT scans. Tracer uptake in each tissue was computed as mean ± standard deviation % ID/g (decay-corrected). Unpaired two-tailed Student’s *t* test was used to compare mean values between groups.

Figure 3a shows the tracer uptake in the xenograft in the blk and nblk groups of the NSG/293 T/hPD-1 mice at 4, 24 and 48 h p.i (mean ± SD % ID/g). Tracer uptake in the blk group was significantly less than that in the nblk group at 4, 24, and 48 h, 1.3 ± 0.3 vs. 2.9 ± 0.3 (p = 0.001), 3.2 ± 0.1 vs. 8.2 ± 0.3 (p = 0.001), and 5.5 ± 0.1 vs. 14.8 ± 1.2 (p = 0.005), respectively. This represents a 100–200% increase in xenograft tracer uptake in the nblk group compared to the blk group for all time points shown. Figure 3b shows that the xenograft-to-muscle ratio (XMR) at 24 and 48 h p.i. was significantly greater in the nblk group compared to the blk group, 7.0 ± 0.7 vs. 3.4 ± 0.9 (p = 0.002) and 15.5 ± 1.4 vs. 4.7 ± 0.6 (p < 0.001), respectively. Figure 3c shows the biodistribution study results of the tracer after the 48 h microPET-CT scan. The xenograft tracer uptake (mean ± SD % ID/g) was significantly greater in the nblk group (17.0 ± 1.6) compared to the blk group (5.7 ± 1.3), p = 0.001. The XMR based on *ex vivo* organ cpm’s was significantly greater in the nblk group (16.2 ± 2.1) compared to the blk group (5.1 ± 1.3), p = 0.003. The tracer uptake in spleen in the NSG/293 T/hPD-1 mice was comparable to that in the xenograft, and was significantly greater in the nblk group (17.5 ± 1.6% ID/g) compared to the blk group (5.7 ± 1.3% ID/g), p = 0.001 (Fig. 3c).

### H&E and Immunofluorescence of Xenograft in NSG/293 T/hPD-1 Mice

**Figure 4:**
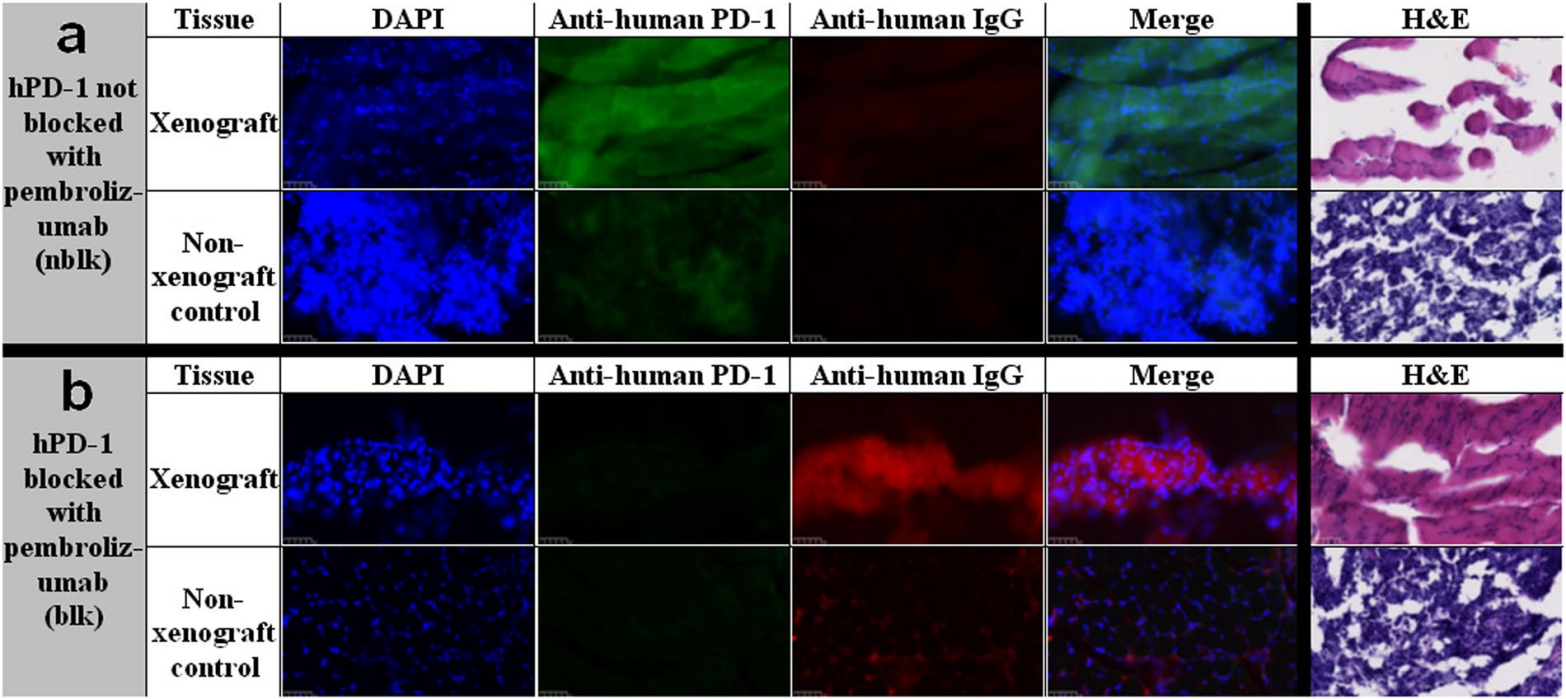
Visualization of hPD-1 and pembrolizumab in tissue sections. (**a**) Representative immunofluorescence (IF) and hematoxylin and eosin (H&E) stains of the NSG/293 T/hPD-1 xenograft and contralateral non-xenograft control tissue from a hPD-1 non-pre-blocked (with non-radioactive pembrolizumab) mouse (nblk). DAPI (blue), anti-human PD-1 (green), and anti-human IgG (red) staining. Zoom 40×. White scale bar in lower left-hand corner of each image represents 50 microns. (**b**) Representative IF and H&E stains of the NSG/293 T/hPD-1 xenograft and contralateral non-xenograft control tissue from a hPD-1 pre-blocked mouse (blk).

Representative images of immunofluorescence and H&E sections of the xenograft and contralateral normal tissue from nblk and blk NSG/293 T/hPD-1 mice are shown in Fig. 4a and b, respectively. H&E (last column of both subfigures) revealed the mostly eosin-staining xenograft tissue compared to the mostly hematoxylin-staining contralateral normal tissue (Fig. 4a,b). From left to right, Fig. 4a,b show immunofluorescence for DAPI (nuclear stain, blue), human PD-1 (a marker for the human 293 T/hPD-1 xenograft, green), human IgG (a marker for pembrolizumab, red), and the merge of the three prior images. Immunofluorescence for human PD-1 demonstrated diffuse staining in the xenograft of nblk mice compared to absent staining in the blk mice, confirming the PD-1-blocking effect of pembrolizumab. In the contralateral normal tissue, nblk mice had minimal/autofluorescence staining compared to absent staining in the blk mice. Immunofluorescence for human IgG demonstrated minimal/autofluorescence staining in the xenograft of nblk mice compared to marked staining in the blk mice, confirming the presence of pembrolizumab in the blk group. In the contralateral normal tissue, nblk mice had absent staining whereas blk mice had moderate staining, again confirming the presence of pembrolizumab in the latter group.

### ^64^Cu-pembrolizumab immunoPET Imaging of Humanized hNSG/A375 Melanoma Mouse Model

**Figure 5:**
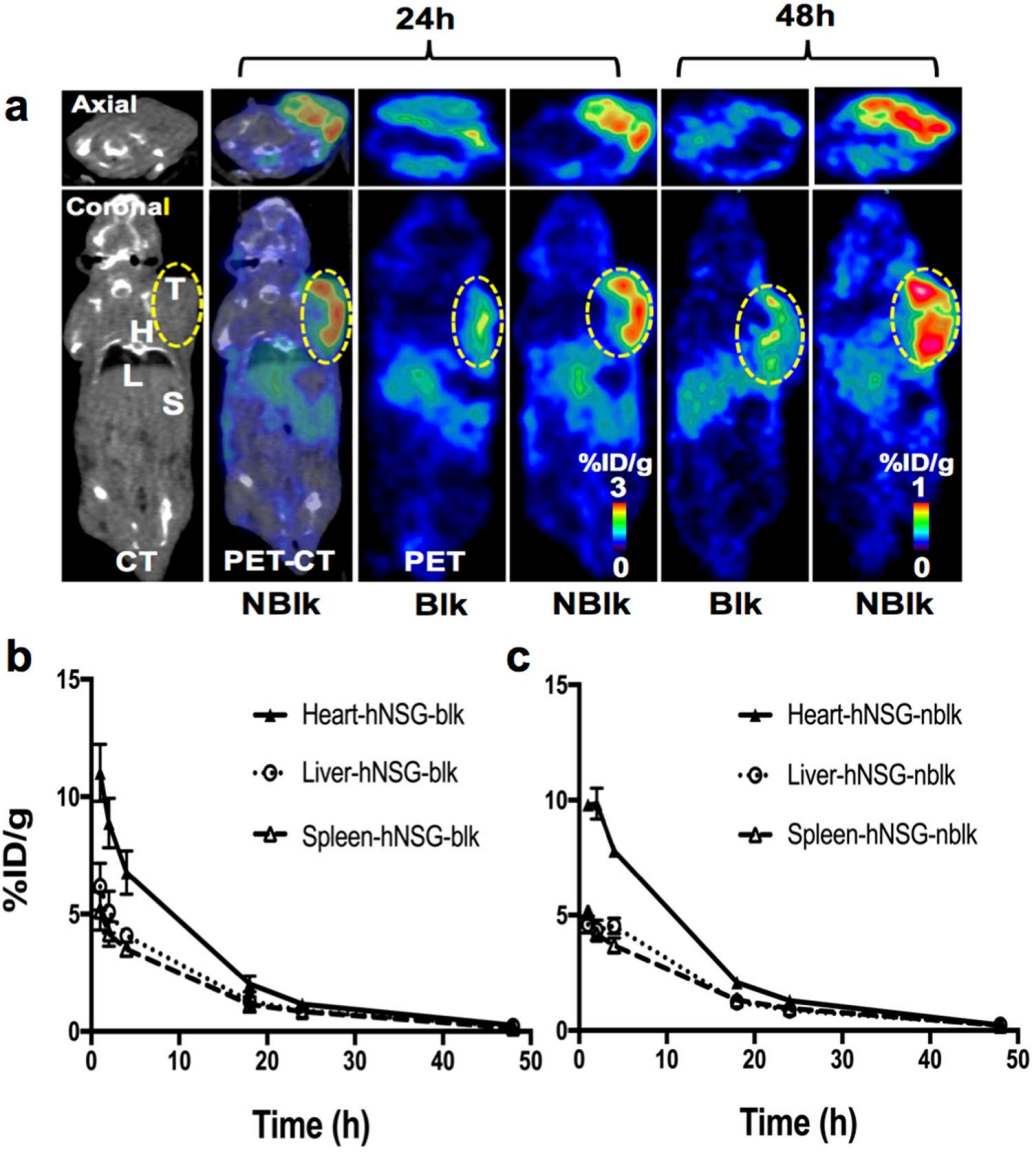
PET-CT image showing ^64^Cu-pembrolizumab immunoPET in hNSG/A375 mouse model. (**a**) Representative PET-CT axial and coronal images displayed at 24 and 48 h post-injection of tracer (7.4 MBq/200 μL) in hNSG/A375-blk, and hNSG/A375-nblk mice. *T* tumor, *H* heart, *L* liver, *S* spleen. (**b**) Time-activity plots of ^64^Cu-pembrolizumab activity residence over time post-injection in clearance organs (*H*, *L*, and *S*) in both groups. Regions of interest were drawn around the *H*, *L*, and *S* corresponding to the images in a. The PET signals of each of the mouse organs are computed as mean ± standard deviation % ID/g at each time point over 1–48 h post-injection. Data are partial volume- and non-decay-corrected.

Figure 5a shows representative axial and coronal immunoPET images (at 24 h and 48 h p.i. of the tracer) of humanized hNSG/A375 mice bearing the melanoma in the left shoulder, with important organs (heart, liver, and spleen) delineated on the representative microCT and microPET-CT co-registered images. These immunoPET images clearly show that the ^64^Cu-pembrolizumab tracer specifically binds to the hPD-1 protein expressed on a subpopulation of human TILs homing to the tumor microenvironment. The marked difference in PET signals (non-decay corrected) between the tumor and background (i.e. muscle) is apparent in the 24 and 48 h p.i. scans. Compared to the nblk mice that did not receive non-radioactive pembrolizumab prior to the scan, the blk mice had significantly decreased tracer tumor uptake at both 24 h (1.8 ± 0.2 vs. 1.4 ± 0.2% ID/g, p = 0.04) and 48 h (0.44 ± 0.01 vs. 0.37 ± 0.01% ID/g, p < 0.001).

Figure 5b,c display the clearance profile of the tracer (non-decay corrected) from heart, liver, and spleen in the blk and nblk groups, respectively. ROI measurement of immunoPET indicated that the PET signal obtained from the spleen and tumor was high due to the hPD-1-expressing lymphocytes present in these sites. For example, at 24 h p.i., the tracer residence (% ID/g ± SD) in the hNSG/A375 mice heart/liver/spleen in the nblk and blk groups was 1.62 ± 0.19/1.10 ± 0.09/1.18 ± 0.10 and 1.16 ± 0.21/0.88 ± 0.05/0.85 ± 0.07 (p = 0.02, p = 0.01, and p = 0.001), respectively. By 48 h p.i., most of the organs in each group had less than 0.3% ID/g of the immunoPET tracer remaining. Supplementary Video S3 is a three-dimensional microPET-CT visualization of hPD-1-expressing tumor-infiltrating lymphocytes in the tumor microenvironment of hNSG/A375 melanoma-bearing mice, at 24 hours post-injection of ^64^Cu-pembrolizumab.

### Dosimetry

Dosimetry (ROI, and *ex vivo* biodistribution) was computed from the biodistribution data of the hNSG/A375 mouse model. The absorbed radiation dose estimates corresponding to human organs for the administration of ^64^Cu-pembrolizumab are summarized in Table 1. The area under the curve of each organ’s activity was computed using the trapezoidal model in Prism software (La Jolla, CA), for both PET/CT-based and *ex vivo* biodistribution data. The PET/CT ROI method revealed that the organs with the highest radiation burden (μSv/MBq) in the blk and nblk groups were the liver (35.2 ± 1.2 and 37.6 ± 2.2, respectively) and heart (17.1 ± 1.5 and 17.8 ± 3.5, respectively). The *ex vivo* immunoPET biodistribution profiles at 1, 12, 24, and 48 hours post-injection in the hNSG/A375 mouse model are shown in Supplementary Fig. S4. The tracer uptake in each tissue was computed as mean ± standard deviation % ID/g (non-decay-corrected). This *ex vivo* method revealed that the organs with the highest radiation burden (μSv/MBq) in the blk and nblk groups were the liver (32.9 ± 3.6 and 34.2 ± 3.4, respectively) and red marrow (18.6 ± 0.9 and 24.1 ± 0.3, respectively). Human equivalent doses (μSv/MBq) were predicted for the blk and nblk groups, and revealed the liver to be the dose-limiting organ in both groups. The dosimetry evaluation from the PET/CT ROI-based and *ex vivo* biodistribution approaches resulted in comparable human equivalent liver doses ranging from 33–38 µSv/MBq. In the blk and nblk groups, the effective dose (μSv/MBq) was 4.3 ± 0.03 and 4.5 ± 0.3, respectively, and the effective dose equivalent value (μSv/MBq) was 2.4 ± 0.1 and 2.5 ± 0.3, respectively. The annual dosimetry of an adult human male receiving pembrolizumab prior to ^64^Cu-pembrolizumab was found to be 1.42 ± 0.05 GBq whereas it would be 1.33 ± 0.08 GBq in patients receiving only the tracer.

**Table 1:** The absorbed radiation doses (corresponding to each organ in humans) for the administration of ^64^Cu-pembrolizumab were estimated with the OLINDA/EXM v 1.2 software (Vanderbilt University, Nashville, TN).

Organ	Total Dose (µSv/MBq)			
	PET/CT images		*Ex vivo* biodistribution	
	Blk	Nblk	Blk	Nblk
**Adrenal glands**	1.89 ± 0.028	2.005 ± 0.12	2.856 ± 0.12	3.38 ± 0.319
**Brain**	0.072 ± 0.002	0.075 ± 0.001	0.422 ± 0.02	0.615 ± 0.041
**Breasts**	0.502 ± 0.002	0.53 ± 0.038	0.867 ± 0.036	1.076 ± 0.11
**Gallbladder wall**	3.24 ± 0.084	3.45 ± 0.197	3.71 ± 0.247	4.14 ± 0.438
**LLI wall**	0.388 ± 0.01	0.405 ± 0.009	1.173 ± 0.06	1.526 ± 0.127
**Small intestine**	0.694 ± 0.003	0.733 ± 0.031	6.14 ± 0.704	6.88 ± 1.462
**Stomach wall**	0.932 ± 0.0003	0.977 ± 0.046	3.256 ± 0.669	3.826 ± 0.967
**ULI wall**	0.928 ± 0.011	0.985 ± 0.049	5.03 ± 1.18	7.286 ± 1.992
**Heart wall**	17.05 ± 1.484	17.75 ± 3.464	6.51 ± 0.4	7.606 ± 1.377
**Kidneys**	1.335 ± 0.021	1.41 ± 0.07	13.166 ± 0.602	17.866 ± 4.834
**Liver**	35.15 ± 1.202	37.55 ± 2.192	32.866 ± 3.617	34.166 ± 3.35
**Lungs**	1.045 ± 0.007	1.105 ± 0.077	10.383 ± 0.748	12.036 ± 1.908
**Muscle**	1.825 ± 0.049	1.905 ± 0.049	2.12 ± 0.156	2.59 ± 0.33
**Ovaries**	0.502 ± 0.008	0.526 ± 0.015	1.413 ± 0.075	1.81 ± 0.181
**Pancreas**	1.735 ± 0.021	1.83 ± 0.098	6.123 ± 0.751	8.36 ± 2.314
**Red marrow**	0.621 ± 0.0004	0.655 ± 0.032	18.566 ± 0.85	24.133 ± 0.251
**Osteogenic cells**	0.5 ± 0.003	0.526 ± 0.023	10.373 ± 0.44	13.6 ± 0.1
**Skin**	0.339 ± 0.002	0.357 ± 0.015	0.594 ± 0.024	0.8 ± 0.114
**Spleen**	6.705 ± 0.289	6.075 ± 0.572	13.5 ± 3.377	18.766 ± 7.479
**Testes**	0.273 ± 0.009	0.284 ± 0.004	0.464 ± 0.034	0.638 ± 0.067
**Thymus**	0.704 ± 0.021	0.739 ± 0.062	1.173 ± 0.049	1.46 ± 0.165
**Thyroid**	0.345 ± 0.01	0.36 ± 0.01	0.691 ± 0.038	0.917 ± 0.08
**Urinary Bladder Wall**	0.4 ± 0.01	0.418 ± 0.009	0.784 ± 0.047	1.037 ± 0.101
**Uterus**	0.473 ± 0.008	0.495 ± 0.014	1.16 ± 0.059	1.523 ± 0.191
**Total Body**	2.015 ± 0.007	2.125 ± 0.106	3.08 ± 0.095	3.693 ± 0.332
**Effective Dose Equivalent**	4.27 ± 0.028	4.46 ± 0.353	8.65 ± 0.055	10.653 ± 1.308
**Effective Dose**	2.37 ± 0.056	2.515 ± 0.134	6.413 ± 0.085	7.69 ± 0.624
**LLI: Lower large intestine, ULI: Upper large intestine**				

The values were determined via two biodistribution methods: PET/CT ROI-based and *ex vivo*. Both methods revealed the liver to be the dose-limiting organ across both mouse groups. Data are presented as mean ± standard deviation.

## Discussion

We previously presented the development of a ^64^Cu-pembrolizumab immunoPET tracer, including synthesis, serum stability, and immunoreactivity^18^. In the current report, we have validated the ^64^Cu-pembrolizumab tracer in two different mouse models, one bearing a hPD-1-expressing 293 T stable cell line xenograft (NSG/293 T/hPD-1) and the other bearing a human A375 tumor that does not express hPD-1 but in which some infiltrating TILs do express hPD-1 (hNSG/A375). The tracer quality was of high immunoreactivity and we were able to visualize the PET signal clearly from hPD-1-expressing cells in both mouse models. Moreover, the PET signal in the hPD-1-expressing TILs present in the non-hPD-1-expressing melanoma contrasted well against clearance organs and background tissues.

The immunohistochemistry (IHC)-based assay determination for hPD-L1 expression to predict response to anti-hPD-1 therapy varies among cancer types. For example, in NSCLC, there is a directly proportional correlation between hPD-L1 expression and response to anti-hPD-1 therapy. However, this hPDL-1/treatment response correlation is not present in melanoma^19^. Given the adaptive nature of the immune response exerted by tumor cells over time, it is possible that the hPD-L1 expression in the tissue sampled for IHC could increase over the course of the disease^20,21^. We have instead focused on imaging hPD-1 expression directly because it indicates the presence of hPD-1-expressing TILs in the tumor microenvironment. This approach raises the possibility for hPD-1 immunoPET to predict responders to anti-hPD-1 therapy, e.g. immune checkpoint blockade with pembrolizumab or nivolumab. Furthermore, once an appropriately selected patient is started on a hPD-1 blocker, immunoPET could also be used as a noninvasive companion diagnostic to assess treatment response. One caveat of this approach is the controversial role of hPD-1 as both an activation marker, and in the setting of some solid tumors with high hPD-L1 expression, an exhaustion marker, of TILs^22^.

England and colleagues previously reported on the pharmacokinetics and biodistribution of ^89^Zr-Df-pembrolizumab in healthy mice and rats, and in severe combined immunodeficient NSG mice engrafted with hPBMCs^23^. However, animal models bearing human xenografts or tumor cells were not studied using ^89^Zr-Df-pembrolizumab tracer. In the current study, we tested the ^64^Cu-pembrolizumab immunoPET tracer in two different mouse models bearing human-derived cells. The 293 T/hPD-1 mouse model served as a positive control because of this non-cancer cell line’s stable expression of hPD-1, and the hNSG/A375 melanoma model served as a representative cancer model in which hPD-1-expresing TILs home to the tumor microenvironment. Furthermore, we studied two groups within each mouse model, pre-blocking hPD-1 with non-radioactive pembrolizumab (blk) and not pre-blocking (nblk), in order to demonstrate the specificity and increased immunoPET tracer tumor (or xenograft) targeting in the nblk group. Although both models show clear immunoPET contrast with respect to tumor (or xenograft) uptake compared to background tissue, the tumor (or xenograft) image patterns are very different. For example, tracer uptake in the NSG/293 T/hPD-1 model (Fig. 2a) is more uniform due to stable expression of the hPD-1 receptor in the xenografted cells. However, in hNSG/A375 tumor mouse model, the tracer uptake is heterogeneous (Fig. 5a), likely illustrating the variable infiltration of hPD-1-expressing TILs that is dependent on the melanoma microenvironment, e.g. the distribution of permeable blood vessels. Further, the hPD-1 signal from these TILs decreases as they likely become exhausted and their hPD-1 receptor relocates to macrophages via FcγRIIb receptor binding^22^.

We predicted the safe human dose that can be injected annually into a patient without surpassing the FDA-allowed dose limits. We performed dosimetry in blocking and non-blocking groups within the hNSG/A375 melanoma mice because this model mimics the situation in which hPD1-expressing TILs invade the tumor. In clinical practice, it is common to administer a non-radioactive antibody to pre-block the spleen from the radiotracer. For example, non-radioactive ibritumomab, a monoclonal antibody targeting CD20, was administered prior to ^90^Y-ibritumomab tiuxetan radioimmunotherapy in relapsed B-cell non-Hodgkin’s lymphoma^24^. This reduces immunoPET tracer splenic accumulation during therapy, allowing more tracer molecules to be available to effectively bind the target site. Likewise, we administered a sufficient pre-dose of non-radioactive pembrolizumab to block off-target hPD-1 binding in multiple organs, including in the tumor, to distinguish the specific binding of the ^64^Cu-pembrolizumab tracer.

The annual radiation dose limit to the whole body, gonads, and active blood-forming organs, and lens of the eye is 50 mSv; and for a single study is 30 mSv^25^. The dose absorbed by all other organs should not exceed 150 mSv annually and 50 mSv for a single study^25^. With respect to ^64^Cu-pembrolizumab tracer, the liver was the dose-limiting organ in both the blk and nblk groups. This is possibly because of its interaction with hepatic Kupffer cells^26^. Also, a small unstable fraction of the ^64^Cu that leaks out from the DOTA chelate may undergo trans-chelation in the liver; however, because the 12.7-hour half-life of the radioisotope is far shorter than the 3–4 week half-life of its bound antibody, the concern for prolonged hepatic radiotoxicity is mitigated^27^.

In the case of 2-deoxy-2-[^18^F]-fluoro-D-glucose ([^18^F]-FDG), the limiting dose in adults is 0.13 mSv/MBq, or 384 MBq per scan^28^. In contrast, the ^64^Cu-pembrolizumab scan dose would be much less, such that the predicted human dose should lower the absorbed dose in critical organs. In a future translational application of this tracer to the clinic, patients would first receive non-radioactive pembrolizumab, followed by ^64^Cu-pembrolizumab. In such a study, we would plan to inject 185 ± 10 MBq of tracer per scan, which would allow for multiple immunoPET scans per year without exceeding the annual dose limit.

Previous anti-hPD-1 immunotherapy studies in NSG mouse models^23,29^ and humans^23,29^ have demonstrated the infiltration of hPD-1-expressing TILs into the tumor and other organs. For example, England *et al*. performed hCD3 and hPD-1 immunohistochemistry to demonstrate infiltration of TILs into the salivary glands of NSG mice engrafted with human PBMCs but not in NSG mice bearing tumors^23^. Using immunofluorescence microscopy, we have demonstrated the pembrolizumab-specific uptake in the 293 T/hPD-1 xenografted mouse model in the nblk compared to the blk group. These immunofluorescence results provide qualitative confirmation of the immunoPET findings that pre-blocking hPD-1 with non-radioactive pembrolizumab (blk) minimizes off-target binding in the xenografted tissue itself.

The results of this study should be interpreted in the context of a limitation. Although we used the 293 T/hPD-1 xenograft model as a positive control to validate our immunoPET tracer with blk and nblk subgroups, we did not use a truly negative control (i.e. a non-tumor cell line that also does not express hPD-1). Overall, the ^64^Cu-pembrolizumab immunoPET tracer is well-suited for imaging hPD-1-expressing TILs because of the decay half-life of ^64^Cu compared to the biological half-life of the antibody (12.7 h vs. 3–4 weeks, respectively), as well as the specific binding demonstrated by the blk and nblk groups. Based on the findings in the current study and those from other groups, we anticipate that this tracer has the potential to detect even small quantities of hPD-1-expressing TILs in the tumor microenvironment, which may otherwise be difficult to directly measure. Moreover, the ^64^Cu-pembrolizumab tracer is more specific with respect to imaging hPD-1-expressing TILs when compared to ^18^F-FDG because of its targeting to hPD-1. Additionally, this tracer could offer a non-invasive method to predict responders to immune checkpoint blockade therapy as well as monitor treatment response, as opposed to the current clinical practice of IHC, which requires tissue and may be vulnerable to under-sampling during biopsy^20^.

## Conclusions

Using two mouse models in which the engrafted tissue either expressed hPD-1 or attracted infiltrating hPD-1-expressing lymphocytes, we have shown that the ^64^Cu-pembrolizumab tracer has specific targeting. This is the first report of human dosimetry of the ^64^Cu-pembrolizumab tracer. The liver was found to be the dose-limiting organ using PET/CT ROI-based and *ex vivo* dosimetry across both mouse groups. The predicted annual human dose of this tracer is well within the FDA-accepted limits.

## Materials and Methods

### Antibodies, Radioisotope, and Reagents

Pembrolizumab (25 mg/mL, Keytruda®; Merck & Co., Kenilworth, NJ), a humanized monoclonal immunoglobulin IgG4 antibody directed against human PD-1 (hPD-1), was purchased from Stanford University Hospital Pharmacy (Stanford, CA). The radioisotope chelator *N*-succinimidyl-DOTA (NHS-DOTA) was purchased from Macrocyclics (Dallas, TX). ^64^Cu CuCl_2_ was purchased from the University of Wisconsin (Madison, WI). All other experimental reagents were obtained from Sigma-Aldrich (St. Louis, MO) unless otherwise stated.

### Cell Lines and Instruments

The hPD-1 stably-expressing 293 T human embryonic kidney cell line was purchased from Crown Biotech (San Jose, CA). These cells were incubated with 3 ng/mL of puromycin in Dulbecco’s modified Eagle medium (DMEM) for epidermal growth factor receptor (EGFR)-positive cell selection. To confirm hPD-1 expression, fluorescence-activated cell sorting (FACS) was performed. For tumor implantations in mice, the A-375 melanoma cell line was purchased from American Type Culture Collection (ATCC® number CRL-1619, Manassas, VA) and maintained in DMEM. DMEM was supplemented with 10% fetal calf serum, 2 mmol/L glutamine, 100 units/mL penicillin, 100 μg streptomycin, and 0.25 μg/mL fungizone. All media and additives were obtained from Invitrogen Corporation (Carlsbad, CA). High-performance liquid chromatography (HPLC) was performed on an HPLC-Ultimate 3000 (Thermo Scientific, Waltham, MA) with a size-exclusion chromatography (SEC)2000 LC column (300 × 7.8 mm) with a 5-μm, hydrophilic-bonded silica support of 400-Å pore size (Phenomenex, Torrance, CA) with an ultraviolet detector and an online radioactivity detector. An instant thin-layer chromatography strip (Biodex Medical Systems, Shirley, NY) with saline as mobile phase was employed as an additional quality test for purity of the tracer.

### Synthesis of DOTA-pembrolizumab and ^64^Cu-DOTA-pembrolizumab

Pembrolizumab antibody was purified by Phenomenex SEC. 2000 HPLC with phosphate buffer solution (PBS, pH 8.0) and buffer exchanged with 1 M HEPES and 0.1 M Na_2_CO_3_ (pH 8.5 ± 0.5) and concentrated to ∼5 mg**/**mL using a Vivaspin 30-kDa centrifugal filter (Thermo Fisher Scientific, Waltham, MA). Coupling conditions and detailed experimental procedures of DOTA-pembrolizumab have been reported^18^. The number of chelators (c) coupled per antibody (a) i.e., c/a, was estimated with matrix-assisted laser desorption/ionization time-of-flight mass spectrometry (MALDI-TOF-MS) by comparison of unmodified pembrolizumab to DOTA-pembrolizumab^18^.

### *In vitro* Live Cell-Binding Assay of DOTA-pembrolizumab

DOTA-pembrolizumab was compared with unmodified pembrolizumab in hPD-1-expressing 293 T cells, and analyzed by FACS. Briefly, pembrolizumab and DOTA-pembrolizumab were diluted to 1 nM solution in FACS buffer (Dulbecco’s PBS). Staining was performed in 96-well microtiter plates (V-bottom). The experimental procedure is described in detail in the Supplementary section. Data were analyzed by FlowJo FACS analysis software version 10.1 (Tree Star, Ashland, OR).

### Synthesis of ^64^Cu-pembrolizumab

A reaction vial containing 0.5 mg of DOTA-pembrolizumab (100 μL) and ^64^CuCl_2_ isotope (260–270 MBq; 750 μL) were mixed (pH, 5.5 ± 0.5 adjusted using 1 M Na_2_CO_3_) and incubated at 37 °C. Ethylenediaminetetraacetic acid (EDTA, 0.1 M, pH 8.0), was added to the reaction vial after 60 min, to achieve a final concentration of 10 mM for 15 min to scavenge unchelated ^64^Cu in the reaction mixture. The ^64^Cu-pembrolizumab was purified by SEC-2000 HPLC in PBS buffer [0.1 M NaCl, 0.05 M sodium phosphate (pH 7.4)] at a flow rate of 1 mL/min. The purified tracer was collected at 8 ± 2 min, corresponding to unmodified pembrolizumab and concentrated to ∼5 mg/mL (by UV 280 nm) using a Vivaspin, 30-kDa cutoff centrifugal filter.

### Quality of ^64^Cu-pembrolizumab: Purity, Immunoreactivity, and Stability

Radio-thin layer paper chromatography (R-TLC) and SEC. 2000 radio-HPLC were performed to assess the tracer quality. Immunoreactivity and serum stability were carried out as per published methods described elsewhere^30,31^. The ^64^Cu-pembrolizumab tracer serum stability was assayed with normal human serum (Sigma Aldrich, St Louis, MO) and incubated at 37 °C. Briefly, tracer and serum were mixed for 6 h. After 6 h, 100 μL was aliquoted and analyzed by radio-HPLC and the eluent was collected in 1-mL fractions and counted in a gamma counter. Percent activity of each fraction was computed to measure tracer stability in serum. Tracer pyrogenicity and sterility tests were performed as per previously published procedures^32^.

### Animal Studies

Animal studies were approved by the *Administrative Panel on Laboratory Animal Care* (APLAC) at Stanford University. NOD.Cg-*Prkdc*^*scid*^*Il2rg*^*tm1wjl*^/SzJ mice (NSG) were purchased from the Jackson Laboratory (Bar Harbor, ME) and maintained in-house in an accredited facility. The average weight of the NSG mice was 23.0 ± 4.0 g. Humanized NSG mice (hNSG/A375) were developed as per the methods published elsewhere^18,33^ and utilized for the immunoPET imaging study. Briefly, human peripheral blood mononuclear cells (hPBMCs) were isolated from normal human blood and tested by FACS for the expression of hCD45- and hPD-1-positive lymphocytes. 5 × 10^6^ hPBMCs were injected via tail vein into NSG mice bearing a human A375 melanoma in the left shoulder, to create hNSG/A375 mice. 293 T/hPD-1 cells were xenografted into the left shoulder of NSG mice, to create NSG/293 T/hPD-1 mice. Furthermore, we studied two groups within each mouse model, pre-blocking hPD-1 with non-radioactive pembrolizumab (blk) and not pre-blocking (nblk), to demonstrate the specificity and increased immunoPET tracer hPD-1 targeting in the nblk group. The blk subgroups received a sufficient pre-dose of non-radioactive pembrolizumab (4 mg/kg) to block the majority of hPD-1 receptors. Supplementary Fig. S5 summarizes the four groups evaluated in this study.

Two groups of hNSG/A375 mice (hNSG/A375-blk and hNSG/A375-nblk, *n* = 4/group), received ^64^Cu-pembrolizumab (200 μL, equivalent of 7.4 ± 0.4 MBq dose, 20–25 μg of DOTA-pembrolizumab) via tail vein injection. After radiotracer administration, microPET-CT imaging was performed on a Siemens Inveon small-animal multimodality PET-CT system (Preclinical Solutions; Siemens Healthcare Molecular Imaging, Knoxville, TN) at the Stanford Small Animal Imaging Center. The PET-CT scanning was performed (microPET default settings with energy window of 350–650 keV) at the following time points after the tracer injection: 1, 2, and 4 h, each for 3-min duration; 18 and 24 h, each for 5-min duration; 24 h for 10-min duration; and 48 h for 15-min duration.

The images acquired from microPET-CT were reconstructed with the two-dimensional ordered-subset expectation maximization (OSEM 2D) algorithm^30^. Regions of interest (ROIs) of selected organs (spleen, heart, liver, tumor, and muscle) were computed using Inveon Research Workplace software (Preclinical Solutions; Siemens Healthcare Molecular Imaging, Knoxville, TN). ROI volume was converted to radioactivity concentration in counts per minute (cpm) by using a predetermined conversion factor. The percent injected dose per gram of tissue (% ID/g) was estimated by dividing the injected dose by the weight of the organ.

### Biodistribution Study of ^64^Cu-pembrolizumab

Two groups of NSG/293 T/hPD-1 mice (n = 3 blk, and n = 5 nblk) were xenografted with hPD-1-expressing 293 T cells. Each mouse was implanted with 2 × 10^6^ cells in a volume of 100 μL of single-cell suspension containing Matrigel/293 T/hPD-1 in the left shoulder. For the NSG/293 T/hPD-1 mice, the subcutaneous tumor height (*h*), width (*w*), and length (*l*) were measured by caliper, and the volume was estimated as an ellipsoid using the formula V = π*h*w*l/6; for the hNSG/A375 mice, the height was estimated as *h* = l*2/3 and the volume was calculated per the aforementioned formula^34^. Mice with 293 T or A375 engraftment volumes ranging from 300–400 mm^3^ were used for the imaging and biodistribution studies. The ^64^Cu-pembrolizumab tracer (200 μL, corresponding to 7.4 ± 0.4 MBq, 20–25 μg) was administered in all four groups of mice (NSG/293 T/hPD-1-blk, NSG/293 T/hPD-1-nblk, hNSG/A375-blk, and hNSG/A375-nblk), which were sacrificed 48 h after the microPET-CT scan. Animals were euthanized by CO_2_ gas asphyxiation for *in vivo* biodistribution studies. *Ex vivo* biodistribution studies were performed separately using the hNSG/A375-blk and hNSG/A375-nblk groups (n = 3–4/group), at four different time points (1, 12, 24, and 48 hours p.i.), and the mice were sacrificed to excise the organs to predict human dosimetry. Organs were removed, rinsed in PBS, dried in air for 5 min, and weighed and counted in a gamma-counter to determine corresponding radioactivity. The immunoPET tracer dose uptake by each organ was determined by measuring the total number of cpm. Count data were background-subtracted and decay-corrected to the time of injection, and the % ID/g for each tissue sample was calculated.

### Histology and Immunofluorescence

24 hours post-injection of tracer in additional NSG/293 T/hPD-1 mice (blk and nblk), the animals were sacrificed. The left-sided subcutaneous tumor and right-sided normal tissue (control) were excised and placed separately in 10% formalin at 4 °C. Two days prior to tissue sectioning, the tissues were placed in 30% (w/v) sucrose in PBS for cryopreservation. On the day of tissue sectioning, the tissues were placed in optimum cutting temperature (OCT, Fisher Scientific, Waltham, MA) compound and frozen to −20 °C. Using a microtome, tissues were cut into 10 μm-thick sections (for immunofluorescence staining) or 25 μm-thick sections (for routine hematoxylin and eosin [H&E] staining), and the sections were mounted to frosted microscope slides and stored at −80 °C.

Tissue slides were removed from the −80 °C freezer and allowed to equilibrate to room temperature over 20 minutes. The tissues were blocked for 1.5 hours in 10% (v/v) fetal bovine serum and 1% (v/v) DMSO in PBS. The blocking solution was then removed. Alexa Fluor® 488 anti-human CD279 (PD-1) antibody (catalog# 329936, Biolegend, San Diego, CA) was used to detect the tumor tissue and DyLight® 594 Goat Anti-Human IgG Fc (catalog# ab97005, Abcam, Cambridge, MA) was used to detect pembrolizumab. After testing serial dilutions of the antibodies, the optimal dilutions were used. The antibodies were diluted in 10% fetal bovine serum (v/v) in PBS, applied to the tissue, shielded from light, and incubated overnight at 4 °C. The next day, the slides were washed three times with PBS. Cover slips were mounted using VECTASHIELD Antifade Mounting Medium with DAPI (catalog# H-1200, Vector Laboratories, Burlingame, CA). The edges of the cover slip were sealed with clear nail polish. Images (40× zoom) were acquired using a NanoZoomer 2.0-RS whole slide imager (Hamamatsu, Hamamatsu City, Japan) and saved as TIFF files using the NanoZoomer Digital Pathology (NDP) Scan version 2.5 software.

### Dosimetry

The human equivalent dose was calculated from the injected dose activity of each animal organ using the method of Kirschner *et al*.^35^. The tracer uptake in each mouse’s organ of interest was manually defined with ROIs by Inveon Research Workspace software from PET/CT images acquired 1–48 hours p.i. ^64^Cu-pembrolizumab uptake was determined in the following organs that are readily demarcated on visual inspection on the PET/CT images: heart, liver, spleen, muscle, and tumor; a final 3D ROI was generated from multiple 2-dimensional images. For *ex vivo* tissue analysis, 12 organs (blood, heart, lungs, liver, spleen, pancreas, stomach, small intestine, large intestine, kidney, muscle, and tumor) were excised and their cpm’s were determined. The ROI values were derived as the mean tracer uptake (nCi/cm^3^) corresponding to each organ, the *ex vivo* distribution studies were derived as % injected dose/gram, and each of these values was subsequently converted to the human equivalent % injected dose/gram for each organ. The residence times for each organ were calculated using trapezoidal integration. The internal radiation dosimetry for each group was evaluated through the normalized cumulative activities that were calculated, and the equivalent residence times for each organ in each mouse group was entered into OLINDA/EXM 1.2 code^34^. The integral value of time activity per organ (μCi-h/μCi injected) was computed from the human equivalent dose. Human dose estimation was performed with the OLINDA/EXM version 1.2 software (Vanderbilt University, Nashville, TN)^36^.

### Statistical Analysis

Unpaired two-tailed Student’s *t* test was used to compare mean values between groups. A p-value less than 0.05 was considered to be statistically significant. Data are presented as mean ± standard deviation (SD).

### Data Availability

All methods were performed in accordance with the relevant guidelines and regulations by Stanford University.

## Electronic supplementary material

Supplementary file

Supplementary Video S3
