# Supplementary material for: Dosimetry Prediction for Clinical Translation of 64Cu-Pembrolizumab ImmunoPET Targeting Human PD-1 Expression
Source: Sci Rep. 2018 Jan 12;8:633. doi: 10.1038/s41598-017-19123-x (PMC5766550; doi:10.1038/s41598-017-19123-x)

Dosimetry Prediction for Clinical Translation of ^{64}Cu-Pembrolizumab ImmunoPET Targeting Human PD-1 Expression

Arutselvan Natarajan[1], Chirag B. Patel[1], Frezghi Habte[1], Sanjiv S. Gambhir[1,2,3*]

[1]Department of Radiology, School of Medicine, [2]Bioengineering, [3]Materials Science & Engineering, Stanford University

Stanford, CA, United States

Supplementary Information

Immunoconjugate Synthesis

DOTA-pembrolizumab conjugate was prepared by mixing 5 mg/mL of pembrolizumab in 1 M HEPES buffer solution, pH 7.5–8.0 (33µM, 1 mL) and DOTA-NHS 34 µL (10 mM solution in dimethyl sulfoxide [DMSO]) of 10 M excess over pembrolizumab into a 1.5 mL microcentrifuge tube. The DMSO concentration was <2% in all the reaction mixtures. The reaction mixture was incubated for 60 min at 37 °C.

The DOTA-pembrolizumab reaction mixtures were purified from excess DOTA-NHS by SEC2000 HPLC using 0.1 M ammonium acetate buffer (pH 5.5) as the mobile phase eluted at 1 mL/min. The immunoconjugate was concentrated to achieve ~2.5 mg/mL using a Vivaspin, 30 kDa cut off centrifugal filter and stored in 200 µL aliquots in 0.1 M ammonium acetate buffer (pH 5.5) at -20 °C.

Cell Assay

In each well, 100 μL of pembrolizumab or DOTA-pembrolizumab was mixed with 100 μL of cell suspension containing 5×10^5 293T/hPD-1 cells and incubated for 1 h on ice. Cells were washed three times with FACS buffer. After washing, 100 μL of biotin-anti-human antibody (eBioscience, Inc., San Diego, CA) pre-diluted to 1:100 in FACS-buffer was added to each well. The ELISA plate containing cells was kept on ice and incubated for 45 min; afterwards incubated cells were washed three times. Finally, the cells were stained with 100 μL of streptavidin-APC (1:100 dilution, BioLegend, San Diego, CA). Dead cells were identified with propidium iodide staining. Analysis was carried out on a flow cytometer (FACS Aria III, BD Biosciences, San Jose, CA, USA) at the Canary Center for Early Cancer Detection, a Stanford University School of Medicine Core Facility (Stanford, CA).

Immunoreactivity

Briefly, 293T/hPD-1 cells were suspended in microcentrifuge tubes (200 μL) at seven concentrations from 1 to 24 million cells per mL in 1% (w/v) bovine serum albumin in PBS (pH 7.4). Each tube received 100 μL of ^{64}Cu-pembrolizumab (stock solution: 5 kBq/22.5 μg/mL) tracer (n = 2). After addition of the tracer (final volume 300 mL), the cells were gently vortexed and incubated at 37 °C for 2 h. Following incubation, two 100-μL aliquots were removed from each tube to measure for total activity as well as cell-bound activity. Tubes marked for cell-bound activity were centrifuged (300 g for 3 min), and the supernatant was removed to measure cell pellet activity. The blocking experiment was performed simultaneously with the same procedure except with the addition of a 100-fold excess of non-radioactive pembrolizumab 1 h prior to the addition of the tracer. Radioactivity associated with cells was measured with a gamma counter (1470 WIZARD Automatic Gamma Counter; Perkin Elmer, Walthem, MA). The counts per minute (cpm) data were background-corrected and compared with the total activity versus bound activity

MicroPET/CT System

This system is capable of operating both the PET and CT scanners independently, or in combination, with excellent radial, tangential, and axial resolutions higher than 1.5 mm at the center of the field of view of the PET module. CT imaging was performed at 80 kVp and 500 μA, second bed position, half scan 220° of rotation, and 120 projections per bed position with a cone beam micro-x-ray source (50 μm focal spot size) and a $40^{64} \times 40^{64}$-pixel X-ray detector. The data were reconstructed using Shepp-Logan filtering and cone-beam filtered back-projection.

Supplementary Figure Legends

Supplementary Figure S1

Measurement of DOTA chelates per antibody by mass spectrometry. Representative MALDI-TOF MS spectra of pembrolizumab (unmodified, black tracing) and DOTA-pembrolizumab (modified, red tracing). The conjugation reaction was performed with pembrolizumab and DOTA-NHS in a 1:10 ratio. The intensity peaks in the 150 kDa region occurred at 147.621 kDa and 148.015 kDa for pembrolizumab and DOTA-pembrolizumab, respectively. Mass difference between the peaks of pembrolizumab and DOTA-pembrolizumab was 394 Da, which represents approximately one DOTA conjugate per antibody.

Supplementary Figure S2

HPLC, and Radio-HPLC trace. Pembrolizumab (blue, HPLC) and ^{64}Cu-pembrolizumab (red, radio-HPLC) traces demonstrating that the quality of the conjugate and radiotracer, respectively, was greater than >99%.

Supplementary Video S3

Three-dimensional microPET-CT visualization of hPD-1-expressing tumor-infiltrating lymphocytes in the tumor microenvironment of hNSG/A375 melanoma-bearing mice, at 24 hours post-injection of ^{64}Cu-pembrolizumab. **(a)** Pre-blocking with non-radioactive pembrolizumab **(b)** No pre-blocking with non-radioactive pembrolizumab. Yellow arrows indicate the location of the tumor implanted into the left shoulder (please see video online).

Supplementary Figure S4

Biodistribution profiles of immunoPET tracer (1, 12, 24, and 48-hour post-injection) in (a) hNSG/A375 pre-blocked hPD-1 (blk) and (b) hNSG/A375 non-blocked-PD-1 (nblk) mice are shown. Tracer uptake in each tissue was washed in PBS and counted as cpm. These cpm values were computed as mean±standard deviation % ID/g (decay-corrected).

Supplementary Figure S5

The four experimental conditions studied in mouse models. The mouse is laying supine with its abdomen facing the viewer. hPBMC = human peripheral blood mononuclear cell, pembro = pembrolizumab, T=human A375 melanoma xenograft, X=human 293T-PD-1 xenograft.

Supplementary Figure S1. MALDI-TOF MS spectra of pembrolizumab and DOTA-pembrolizumab.

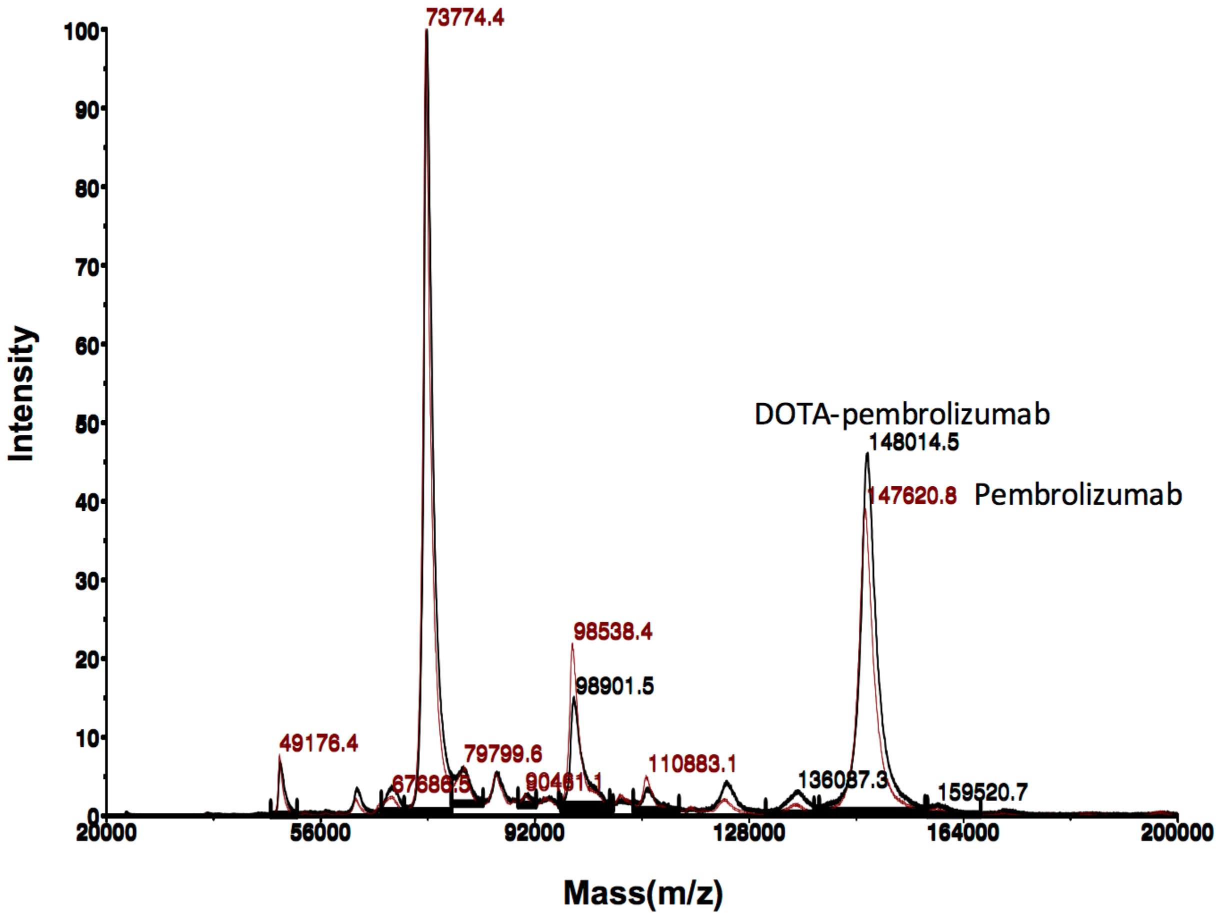

Supplementary Figure S2. HPLC (pembrolizumab) and radio-HPLC (^{64}Cu-pembrolizumab) chromatograms.

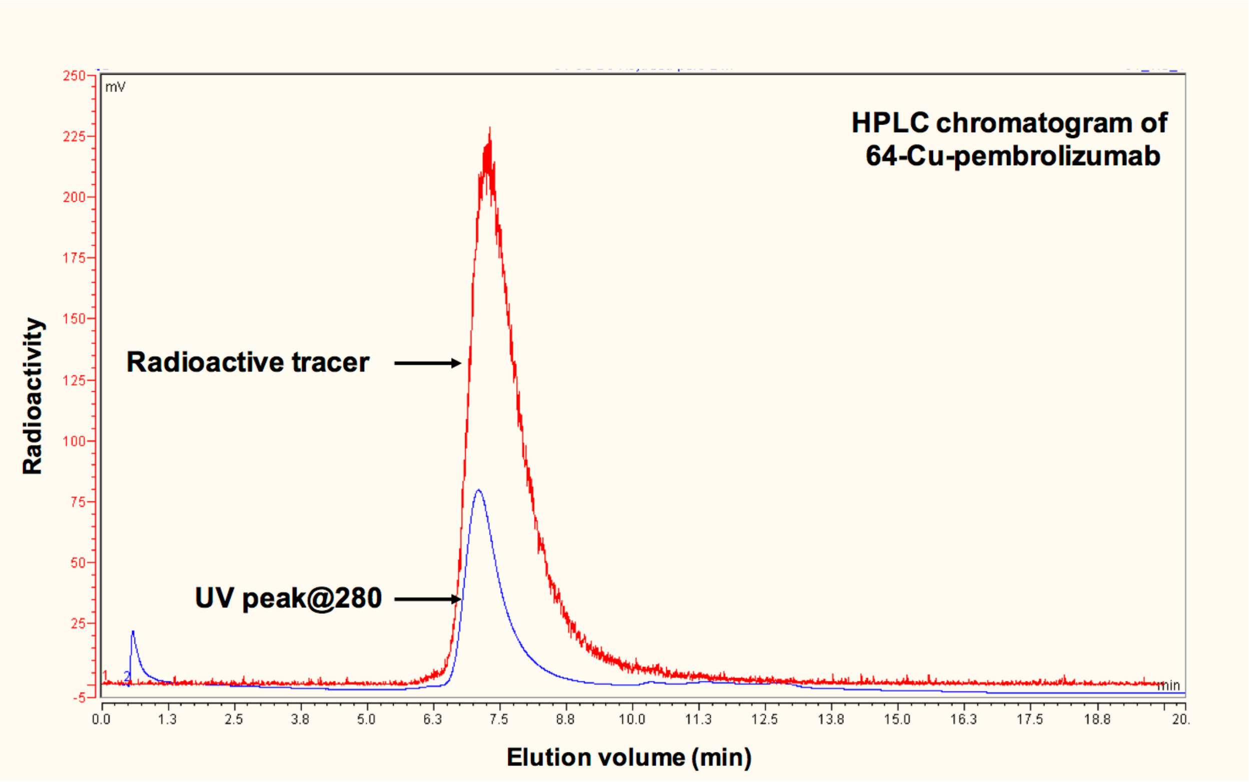

Supplementary Video S3. MicroPET-CT visualization of hPD-1-expressing tumor-infiltrating lymphocytes using ^{64}Cu-pembrolizumab.

Please see video online.

Supplementary Figure S4. Ex vivo biodistribution profiles in hNSG/A375 mouse model

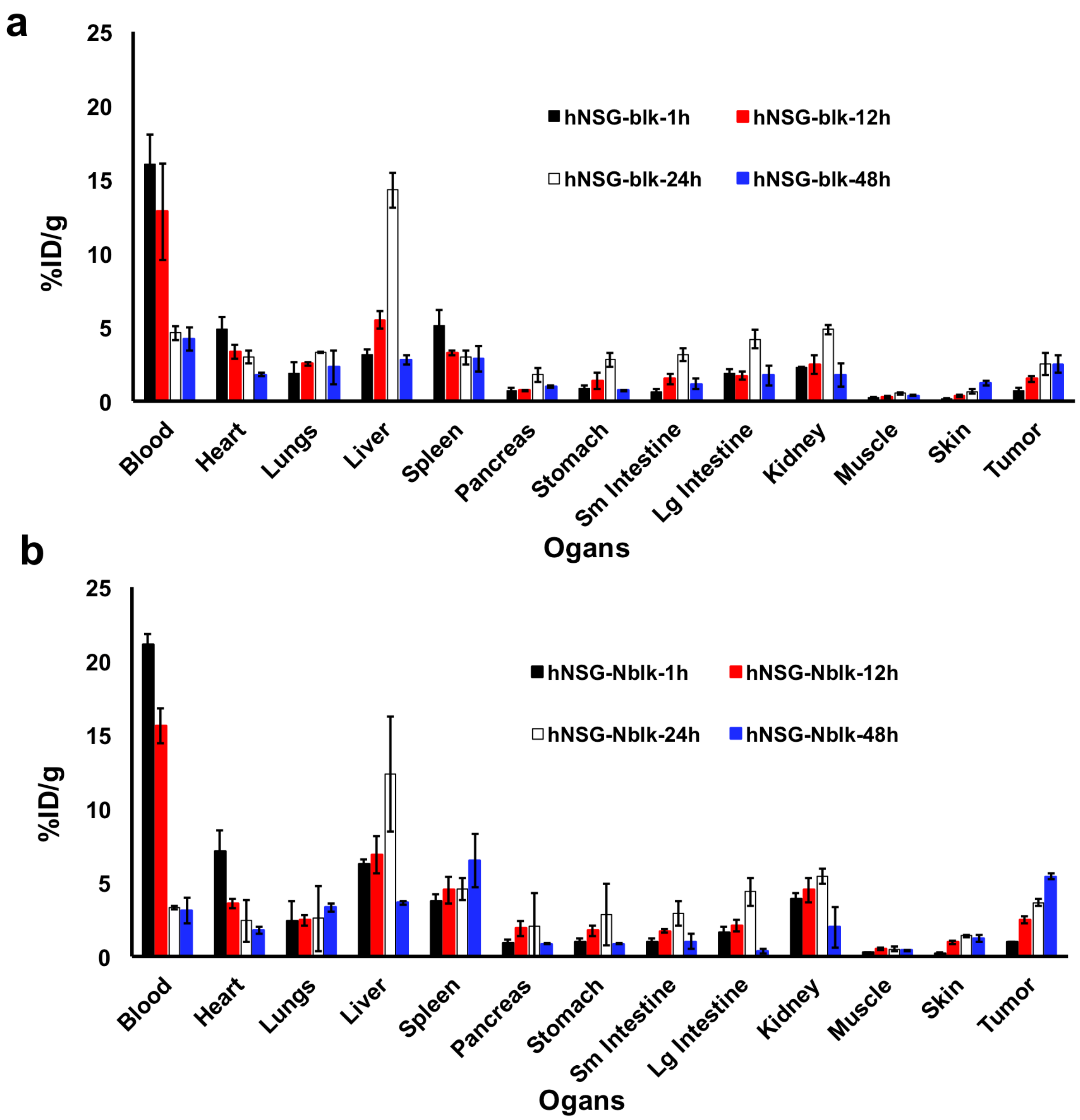

Supplementary Figure S5. Four mouse groups studied with immunoPET

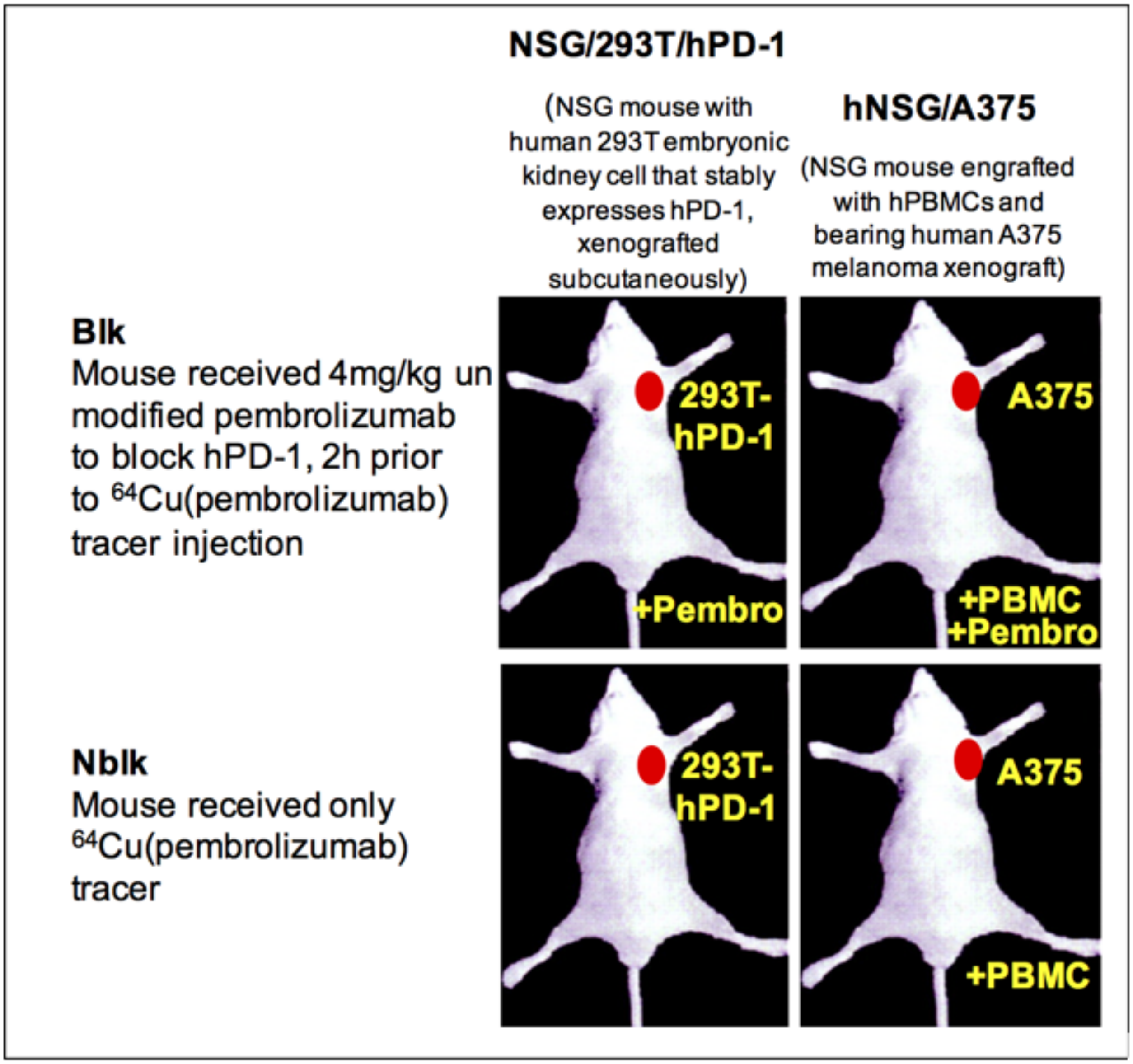

Supplement: Supplementary file 1 — Supplementary file [file 41598_2017_19123_MOESM1_ESM.pdf]
